# Monitoring vaccination coverage: Defining the role of surveys

**DOI:** 10.1016/j.vaccine.2016.06.053

**Published:** 2016-07-29

**Authors:** Felicity T. Cutts, Pierre Claquin, M. Carolina Danovaro-Holliday, Dale A. Rhoda

**Affiliations:** a London School of Hygiene and Tropical Medicine, UK; b World Health Organization Consultant, Lamballe, France; c World Health Organization, Geneva, Switzerland; d Biostat Global Consulting, OH, USA

**Keywords:** Vaccination, Coverage, Surveys, Program monitoring, Health facility surveys

## Abstract

Vaccination coverage is a widely used indicator of programme performance, measured by registries, routine administrative reports or household surveys. Because the population denominator and the reported number of vaccinations used in administrative estimates are often inaccurate, survey data are often considered to be more reliable. Many countries obtain survey data on vaccination coverage every 3–5 years from large-scale multi-purpose survey programs. Additional surveys may be needed to evaluate coverage in Supplemental Immunization Activities such as measles or polio campaigns, or after major changes have occurred in the vaccination programme or its context.

When a coverage survey is undertaken, rigorous statistical principles and field protocols should be followed to avoid selection bias and information bias. This requires substantial time, expertise and resources hence the role of vaccination coverage surveys in programme monitoring needs to be carefully defined. At times, programmatic monitoring may be more appropriate and provides data to guide program improvement. Practical field methods such as health facility-based assessments can evaluate multiple aspects of service provision, costs, coverage (among clinic attendees) and data quality. Similarly, purposeful sampling or censuses of specific populations can help local health workers evaluate their own performance and understand community attitudes, without trying to claim that the results are representative of the entire population. Administrative reports enable programme managers to do real-time monitoring, investigate potential problems and take timely remedial action, thus improvement of administrative estimates is of high priority. Most importantly, investment in collecting data needs to be complemented by investment in acting on results to improve performance.

## Highlights

• High quality community-based vaccination coverage surveys are resource-intensive.
• Other monitoring methods provide useful data for programme managers.
• Health facility-based assessments evaluate multiple aspects of service provision.
• Purposeful community samples give local health workers programmatic insights.
• To be useful, monitoring should lead to action to improve performance.

## 1 Introduction

Vaccination coverage is a widely-used indicator of vaccination programme strengths and weaknesses and of access to health care [1], [2]. High coverage of the first dose of diphtheria-tetanus-pertussis-containing vaccine (DTPCV1) indicates good access to primary health care facilities; by contrast a high proportion of zero-dose children suggest either low access to services or lack of acceptance of vaccination. High dropout between early and final doses of the primary vaccine series may indicate health system barriers to re-attendance, failure to educate mothers of the need to return, or inadequate tracking of children registered at the health facility. Missed opportunities to administer all vaccines scheduled at the same visit (“non-simultaneous vaccination”) may indicate vaccine stock-outs, mistakes in identifying which vaccines are due, reluctance to vaccinate a sick child or to administer multiple vaccines at the same visit, etc. [3], [4], [5]. Monitoring the age at receipt of each vaccine-dose (“timeliness”) helps verify that vaccines are not administered too early, which might reduce vaccine effectiveness, yet as soon as possible after the scheduled age to minimize the time that the child is at risk of infection [1], [6], [7], [8].

Several methods are used to monitor coverage [9] each having advantages and disadvantages (Table 1, adapted from [10]). Electronic vaccination registries can provide continuous data for coverage measurement and for management activities such as monitoring vaccine supply and requisitions and sending vaccination reminders [11], but there are many challenges to their implementation [12], [13]. Most low-income countries rely on paper-based systems to report vaccinations administered and divide by the estimated target population to derive “administrative coverage estimates”. Administrative estimates, however, are often unreliable due to incomplete or inaccurate primary recording of vaccinations, mistakes in compiling monthly summaries of vaccinations, delayed or duplicate reporting and inaccurate estimates of population denominators [1], [14], [15], [16].

Household surveys are often proposed because EPI managers or global partners do not trust administrative reports. Vaccination coverage is measured in the large-scale Multiple Indicators Cluster Survey (MICS) [17] and Demographic and Health Survey (DHS) [18] programmes which use probability sampling methods (i.e. one in which each individual has a known and non-zero chance of being selected) and strict quality control with substantial technical assistance [19]. The Expanded Programme on Immunization (EPI) cluster survey was developed over 30 years ago as a simple non-probability sampling method that could be implemented with little or no technical assistance [20], [21]. Although the EPI survey has been a valuable programme management tool, the use of non-probability sampling and lack of standardized, well-documented quality control procedures may reduce confidence in the results [1], [22], [23]. The 2015 working draft of the EPI cluster survey manual recommends using a probability sample (for which excellent collaboration is needed with the National Statistics or Census Office to obtain the sampling frame and maps of enumeration areas (EAs)), designing the survey and its sample size according to the evaluation goals, conducting appropriately weighted analyses, rigorous quality control, and fully documenting survey design and implementation. This will increase the technical and financial resource needs for vaccination coverage surveys, which should therefore be used judiciously.

In this paper, we discuss issues that affect decisions to undertake household surveys and propose some alternative monitoring methods to answer programme questions at peripheral health system levels. Unless otherwise specified, we use the term household surveys when referring to community-based surveys using probability sampling.

## 2 Common goals of vaccination coverage surveys

In the past, surveys have been conducted frequently to monitor trends in national routine vaccination coverage. By 2013, however, many countries had reached over 85% coverage, beyond which annual increases will be small and DHS or MICS are conducted often enough to monitor changes. A large proportion of countries where coverage is still low are affected by conflict which inhibits both vaccination and the conduct of surveys representing the entire population of the country. Therefore, surveys with a primary goal of measuring routine immunization (RI) coverage at national level will either rarely be needed or not be feasible. By contrast, evaluation of coverage achieved in Supplemental Immunization Activities (SIAs), for example those used to introduce rubella vaccine in developing countries, is becoming more important [24].

Other goals of coverage surveys include comparing coverage in different provinces of a country, evaluating the coverage of vaccines recently introduced into the national immunization programme, and evaluating coverage in certain areas of the country selected e.g. because they have been targeted by specific interventions, or are recovering from natural or man-made disasters. Although data are desirable at the most peripheral levels, household surveys are too time-and resource-intensive to conduct in every district and other monitoring methods are more suitable at district level. One possibility is to conduct surveys that provide imprecise estimates in each district but have adequate size to classify coverage as likely to be very low or very high, and to aggregate the data from each district as a stratified sample to obtain estimates with reasonable precision at the next-higher level e.g. provincial level and with very tight precision at national level. In this document when we say “useful to classify”, we mean a sample too small for precise estimation at the peripheral level, but probably useful for distinguishing very poor and very good performance and also useful for aggregation up to higher levels of hierarchy.

## 3 Are data from coverage surveys accurate?

Coverage estimates from surveys are often trusted more than administrative estimates [16] but, like administrative estimates, their accuracy depends in part on the quality of primary recording of vaccinations. In addition, surveys are subject to other types of information bias, selection bias and sampling error [1], [25].

Selection bias can occur when the list of eligible respondents (the sampling frame) excludes subpopulations (e.g. in conflict settings, or areas with large homeless populations), when field procedures use non-probability sampling methods or substitute a sampled household with one which is easier to reach, or when revisits to households where a respondent was initially absent are not done or unsuccessful. The populations likely to be missed in a vaccination coverage survey are also likely to be missed by vaccination teams, so selection bias most often inflates coverage estimates.

Information bias occurs when a child’s vaccination status is misclassified due to mistakes in the vaccination record, transcription of data from the record, or the guardian’s recall for children without a written record (which may be affected by the way the interviewer asks the questions and the complexity of the vaccination schedule). It can bias results upwards or downwards, in part depending on the degree to which the respondent perceives social pressure to report complete vaccination [26], [27]. The number of vaccines in national schedules in developing countries has more than doubled since 1980 with a consequent increase in the burden of recording on home-based and health-facility-based records. The 2015 WHO EPI manual recommends that when a home-based record is unavailable, documents should be sought at health facilities, but the extent to which this will be feasible and the improvement in quality gained with its implementation in different countries is as yet unknown. Information bias may still occur because documents are missing or contain errors [1].

## 4 What factors affect the time and other resources needed for vaccination coverage surveys?

EPI coverage surveys using probability samples are expected to take at least 12 months between inception and the final report (Table 2). The timeframe may be shorter for a post-SIA survey if data are only needed at national level and only vaccines administered in the SIA are assessed. Surveys should be planned well in advance, and for post-SIA surveys, planning should start concurrently with preparation for SIA implementation. The length of the planning phase depends in part on financing and sub-contracting mechanisms, as well as the time needed to obtain ethical clearance, census data, maps, and resources. The timeframe for the survey must be realistic taking into account the feasibility of ensuring that all field workers follow protocol properly. It is better to work with 30 excellent field workers than 300 poor or mediocre field workers, and to ensure that all teams are well supervised and quality control is rigorous. Factors affecting the human, financial and logistical resources as well as the time needed to implement a survey include (but are not limited to):

• Whether the goals are estimation of coverage (and with what precision) or classification of coverage as probably high, probably low, or intermediate (and with what tolerance of the chance of misclassification). It may be more practical to aim to classify coverage at sub-national levels and roll-up the data to estimate coverage precisely at national level than to try to estimate coverage at each sub-national level.
• For hypothesis testing, how narrow a difference between groups is to be detected, with what power and at what significance level.
• The number of strata. Resource needs increase proportionately to the number of sub-national strata where results are reported. For example, compared to a single national estimate of a given precision (e.g. ±5%), in a country that has 10 provinces, each with 10–17 districts (total of 150 districts in the country), the sample size would increase 10-fold if survey results of precision ±5% are desired in all provinces, and 150-fold if district-level estimates of ±5% are desired.
• The number of age cohorts for which results are desired – e.g. stratifying SIAs that target children up to 15 years to estimate coverage by 5 year age groups increases the sample size compared to a single estimate for all persons under age 15 years. If a survey is already stratified by province, it may be best to aim to obtain data by age-group at national level, where the total sample size rolled-up from all provinces is much higher.
• The number of health topics evaluated in the survey, as sample size requirements vary for different indicators, and the more health topics, the longer the questionnaire. Furthermore, long questionnaires may also affect the quality of the data on each topic due to interviewer and respondent fatigue.

Note especially the implications of evaluating routine vaccination coverage during a post-SIA survey. For the same target precision, the required sample size is higher for routine than for SIA coverage because coverage in the former is usually lower and more heterogeneous. This is compounded by the need to visit more households to find eligible persons because routine coverage is assessed in a much narrower age group than SIA coverage (e.g. see Table 3). Furthermore, the questionnaire is much longer for RI because there are now more than 20 potential vaccine-dose combinations in the routine vaccination schedule to record, and health facilities should be visited if the child’s caregiver cannot furnish a home-based record, whereas SIA vaccination is not recorded in health facilities so such visits are not recommended. Thus, evaluating all routine vaccinations among 12–23 month olds during a post-SIA survey increases resource needs and may delay results of SIA coverage. It will often be best to focus resources on ensuring optimal supervision during the SIA and obtaining timely, high-quality SIA coverage data. One or two questions can be included about prior receipt of the relevant vaccine (e.g. measles-containing vaccine if a measles or measles-rubella SIA is being evaluated) through routine services so as to evaluate whether the SIA reached persons missed by the routine programme.

## 5 How often should coverage surveys be done?

The frequency of surveys depends on how likely it is that results have changed since the last high quality survey in the same area, which in turn relates to the level of coverage in the last survey, the timing of any interventions conducted to increase coverage, and the extent of changes in health system or political context that may affect coverage. It also depends on progress in improving administrative data.

In countries with sustained high routine coverage (e.g. WHO-UNICEF estimates of DTPCV3 85% or above), it becomes more difficult to identify a statistically significant increase – in fact, by chance one could see a fall in the point estimate which could discourage health workers who do not understand sampling error. SIAs are done less frequently than in countries with low RI coverage [24] and nationwide surveys will therefore be needed infrequently. Surveys may be done at sub-national level, e.g. to evaluate interventions in specific areas of the country where coverage had been lower than average.

Countries with coverage below 80% still need to achieve large gains to reach goals such as those in the Global Vaccine Action Plan [2], thus surveys can be useful to monitor trends. Their role may be limited, however, by conflict which affects almost half the countries with median WHO-UNICEF coverage estimates of DTPCV3 below 80% from 2011–2013 [28]. Surveys can help to measure coverage in the more secure areas but trends may be difficult to interpret if different parts of the country are accessible in each survey. When programmes are weak and/or there is marked internal population displacement, documentation of vaccination status is harder to obtain, further reducing the reliability of survey data. Although administrative data are also inaccurate in many of these countries, routine information on programme inputs and process indicators such as number of vaccination sessions held, number of outreach sessions held on time, number of vaccine stock-outs, etc., plus health facility surveys (see below) and rapid convenience monitoring [9] in accessible areas, can provide insights to improve programme performance.

## 6 What alternatives to household surveys are useful for programme managers?

Before considering a new survey, existing data should be reviewed to assess whether there is enough information to take programme decisions. For example, because administrative reports most often tend to over-estimate than under-estimate coverage [16], if administrative estimates show low national coverage of first dose DTPCV, and there is no major under-reporting of vaccination (e.g. if a large private sector does not report) or evidence of a markedly inflated denominator, then coverage is likely to be truly low. It may be more important to investigate and address the reasons for low coverage than to do a survey to verify precisely how low coverage is. Reasons for low access to or use of vaccination services can be assessed by reviewing information on service delivery from reports of supervisory visits, recent programme reviews and supply chain assessments. Formative research such as focus groups and key informant interviews with health workers, community leaders, and community groups provides insights to help local programme managers modify delivery strategies appropriately.

If administrative reports and/or recent surveys show high dropout rates, then reasons should be investigated. Coverage surveys measure the extent of dropout and non-simultaneous vaccination but not their causes and hence do not inform the design of strategies to reduce them (Table 4). Health-facility surveys (including outreach sites) are very useful to assess multiple aspects of service delivery including missed opportunities and their causes [29], [30], vaccine supply chain, tracking activities and health worker knowledge, attitudes and practice about vaccination and safe injections (Table 4). The costs of each aspect of service provision can also be estimated. The human factor in providing vaccination services (absenteeism, quality of training, wrong interpretation of contraindications, etc.) should be carefully assessed. The quality of data recording can be evaluated by comparing data from home-based records reviewed during exit interviews with data from clinic registers for the same children, and comparing register data with reports to higher levels [31], [32]. Although health facility surveys do not provide data that represents the entire population (or even all health facilities, depending on whether facilities are selected purposefully or randomly), and are subject to observer bias if the presence of an interviewer at the health facility affects the way the health workers perform on that day, they often identify problems with health service provision. Results can be considered a “best case” scenario and interventions to improve services undertaken soon after the surveys are completed.

Health facility surveys can be combined with other methods that district health personnel can conduct with minimal external support, such as “100 household surveys” (later termed “75 household surveys”) in which households closest to a health facility are canvassed and vaccination status assessed for children aged 12–23 months together with caretaker and community opinions about vaccination services [29], [33]. Although the 100-household survey is a purposeful sample and is not generalizable to households farther from the health facility, it is useful to determine reasons for incomplete or delayed vaccination among persons with good physical access to health facilities. A similar purposeful sampling method to check vaccination status of all individuals in the target age groups of interest could be implemented in villages far (e.g. >5 km) from a health facility or outreach site. Such “mini-censuses” can be done by local health workers in areas that for common sense reasons are thought likely to have lower coverage. Such censuses should cover the entire population of those areas and not only assess vaccination coverage but also identify and refer infants who are behind on vaccination (and other health needs can be assessed during the same visits). They could be done on a rolling basis to cover many areas over the course of the year. These kinds of community-based activities involving door-to-door visits and community discussions should become part of an ongoing process of community engagement in planning, implementing and monitoring vaccination services. Although not based on random samples and therefore not providing robust estimates of average district coverage, they are valuable learning tools for health workers and provide in-depth information on entire population subgroups which is more easily understood by communities and health workers (since ALL residents of the given areas are canvassed). This should more easily lead to local action to improve services [9].

## 7 Conclusions

Coverage surveys have played an important role in helping countries monitor progress of vaccination programmes, but their role is changing as many programmes have matured. Surveys have an important role to evaluate SIA coverage, where reported coverage is particularly likely to be inaccurate because during SIAs there is little time to check the age or residency of persons attending for vaccination or to record and report vaccinations accurately. For routine vaccination coverage, DHS and MICS will usually be sufficient to monitor trends at national and often also provincial levels.

Donors may desire a coverage survey because they lack confidence in administrative estimates, but the likelihood that accurate and representative results can be obtained through household surveys at reasonable cost needs to be assessed carefully. For example, if major areas of the country are inaccessible due to insecurity then results will not represent the whole country; if there have been known stock-outs of home-based vaccination records, and health facility registers are poorly filled in or difficult to access, then information bias will be difficult to avoid. How the results will help managers at different levels to improve their programmes should be assessed. Surveys can always give “some useful information” but there will often be quicker and cheaper ways for programme managers to get more actionable information, including health facility assessments and purposive sample surveys.

If a coverage survey is done, it is important to define and prioritize objectives clearly for each geographical level, with realistic assessment of the required resources and time for different potential objectives and compromise on the goals of the survey if needed to ensure it will have excellent quality with the available resources. The survey must be implemented according to protocol, with strict quality control of all aspects of sampling, data collection, management and analysis. Results should be translated into information that leads to action, with feedback at all health system levels. The links built with statistics or census offices for surveys should be expanded on, e.g. using population data and maps to improve district-level planning.

At sub-national level, in most instances, simpler methods will be suitable for programme needs. Health facility assessments should be conducted regularly and can be complemented by censuses of vaccination status, knowledge, attitudes and practices among neighbourhoods and villages near to and far from sites offering vaccination. These can be planned, implemented and analyzed by district health staff, thus maximizing the learning experience, and their results are available at the local level very quickly thus facilitating action.

The resources that would be required for household probability sample surveys will often be better invested in supporting locally-led monitoring and acting to strengthen routine reporting systems, especially the primary recording of data, which would improve both administrative estimates and future survey estimates. Most importantly, investment in collecting data needs to be complemented by investment in acting on results to improve programme performance.

## Conflict of interest

The authors declare that they have no conflict of interest.

## Figures and Tables

**Table 1:** Advantages and disadvantages of methods to measure vaccination coverage.

Method	Advantages	Disadvantages
Register-based (electronic)	Can give complete and accurate real-time data on cumulative vaccination status of individual persons and populations Can be used to set appointments and issue reminders and recalls Can be used for vaccine stock control, ordering and accountability Could reduce time spent on paper registers that are widespread in low-income countries and often not used Can facilitate printing or electronic access to home-based vaccination records Can provide data at most peripheral operational level	Need good computer access Need complete birth registry for true denominator Need unique ID number throughout life Need procedures to identify and deal with potential duplicate records If held locally, difficult to track vaccination of migrants If held nationally, feedback/use at local level may be slow Requires adequate funding for proper maintenance Need sufficient, well-trained human resources at each level of the reporting system Need secure procedures to maintain confidentiality Need procedures to avoid losing data Difficult to use to measure coverage in SIAs

Routine reports of vaccinations delivered	Can be simple in conception Continuous information allows monitoring of cumulative coverage through the year and by district/health facility Can be used by local health workers to track coverage, missed opportunities, and dropout rates Usually part of a routine reporting system used for multiple health programmes	Population denominators often inaccurate, especially at local levels Private sector often does not report Exaggeration of doses administered common, especially when linked to performance-based incentives Transcription errors at each health system level when paper-based systems used In SIAs, reports often give inflated estimates due to short time for recording, and vaccination of persons outside the target age group

Community-based surveys	If well-conducted, evaluate coverage in routine services and/or in SIAs Other indicators (eg, missed opportunities, caretaker demographics and knowledge/attitudes) can be assessed although this increases questionnaire length and complexity Can be used to classify coverage e.g. as “probably high”, “probably low” or “indeterminate” in subnational areas and highlight those with lowest coverage Can allow estimation of coverage in specific sub-groups if designed appropriately Involvement of health workers can be training opportunity	Accessibility to populations to survey depends on geographic, climatic and security issues, and high-risk subgroups (e.g., migrants, street children) often missed, compromising representativeness of survey results Small samples give imprecise results; large samples are expensive and field work takes longer In some settings, it may be difficult to obtain accurate ages/dates of birth Accuracy of data depends on adequate survey design, training, supervision, and quality control, as well as availability of vaccination documentation Information bias likely if documents are missing, incomplete or inaccurate – verbal history increasingly difficult as more vaccines included in programmes under a range of different schedules Often subcontracted to private organisation hence health worker training opportunity lost Often long delays until results are known, and survey data relate to birth cohort at least one year prior to survey implementation

Adapted from Table 70-3 in [10].

**Table 2:** Illustrative timeframe for a probability sample household coverage survey.

Stage	Activity	Dates
Planning and survey preparation	Form steering committee and technical subcommittees, identify implementing agency, agree on methods to recruit field coordinators, supervisors and interviewers, agree on use or not of digital technology for data collection, identify technical assistance if required, set up liaison with statistics or census office, order and obtain supplies & identify transport	Months 1–4 (may take longer if Request for Proposals issued for selection of implementing agency, if complex survey design with multiple indicators, depending on ethics committee procedures and timetable, and depending on time needed to obtain and release funding)
Survey design and modification to fit resource availability
Obtain funding for the survey
Obtain ethical review as required
Sample selection (including obtaining enumeration area-EA maps)
Visit health facilities in the areas selected for the survey to explain survey and obtain co-operation
Questionnaire design, pretest and translation
Preparation of digital entry procedures, if used
Pretest household sampling procedures
Preparation of manuals/standard operating procedures
Preparation of training site(s) and materials
Preparation of database

Training	Train field workers and supervisors: household listing, collection of GIS coordinates, conducting interviews, getting data from health facilities, checking completed questionnaires, digital data entry where relevant, ensuring SOPs are followed	Month 5 (longer for large surveys; allow 2 weeks for every 30 field staff being recruited)
Train data entry staff if paper forms are used

Data collection	Mapping and household listing Collection of data from eligible persons with revisits as needed Quality control in the field, including random revisits by supervisors Resolution of queries	Months 6 (if small survey), or 6–8 (for survey with multiple strata)

Data management and analysis	Data double-entry and editing (if paper forms used)	Months 6–7 (small survey) or 6–9 (large survey)
Final data checking and cleaning
Data analysis, produce tables and graphs

Report generation and dissemination	Preparation/review of preliminary report	Months 10–12 (may be sooner if small and focused survey is done)
Prepare final report, with summary of key findings
National feedback and develop action plan
Prepare reports/fact sheets for health workers
Workshops with health workers at sub-national levels

**Table 3:** Sample size and number of households that must be visited in a post-SIA survey with and without inclusion of routine immunization (RI) coverage assessment.

		N (Completed Interviews)				HH (Households to Visit)			
Expected Coverage		Post-SIA Alone	RI DTPCV3 12-23m Width of confidence intervals:			Post-SIA Alone	RI DTPCV3 12-23m Width of confidence intervals:		
SIA	RI	±3%	±3%	±5%	±10%	±3%	±3%	±5%	±10%
95%	90%	708	1554	645	210	1416	7770	3225	1050
95%	85%	708	1989	795	240	1416	9945	3975	1200
95%	75%	708	2676	1020	285	1416	13,380	5100	1425
90%	60%	1036	3291	1203	309	2072	16,455	6015	1545

SIA = Supplemental Immunization Activity; RI = Routine Immunization; DTPCV3 = 3 Doses of Diphtheria Tetanus Pertussis Containing Vaccine.

Notes: This table assumes a post-SIA design effect of 2.0 and an RI design effect of 3.0, as intracluster correlation is expected to be higher for the latter. It also assumes that eligible respondents for post-SIA survey are 9m-15y of age and that it will be necessary to visit two households, on average, to find each eligible and cooperative respondent. Eligible RI respondents are 12–23 m of age and it will be necessary to visit 5 households, on average, to find each eligible and cooperative respondent.

**Table 4:** Choice of methods according to the purpose of monitoring.

Monitoring/evaluation purpose	Cluster survey with probability sampling and excellent quality control			Health facility-based surveys (note: results refer only to persons using the facilities sampled)	Purposeful sampling or censuses of selected populations
National	Every province	Selected provinces/districts
To measure coverage in an SIA at national level	Yes	Yes	No	No	No
To evaluate SIA coverage at national *and* sub-national levels	No	Yes	No	No	No
To measure national routine vaccination coverage	Yes	Yes	No	No	No
To measure routine coverage at sub-national levels	No	Yes, though not feasible in all districts	Yes, for selected areas only	No	Yes, in the areas included, with no sampling error
To classify coverage at sub-national levels	No	Yes	Yes, for selected areas only	Yes, among children who’ve received DTPCV1	Not relevant
To determine if an intervention has successfully increased coverage	Possibly, if sample size adequate (based on baseline and follow-up sample precision)	Yes, if sample size adequate to detect the difference. Especially useful if some areas had the intervention while others did not, and if baseline and post-intervention are available for areas with and without the intervention		Can assess if an intervention has increased coverage of subsequent vaccines among children who have received DTPCV1	Theoretically possible, but probability samples of larger areas usually preferred
To assess dropout, timeliness, missed opportunities	Can measure dropout and missed opportunities but does not assess their health-system causes			Can assess both prevalence and causes	Can assess prevalence in the areas canvassed
To identify reasons for lack of receipt of DTPCV1	Yes if suitable questions added to the survey, ideally informed by prior qualitative research (and need appropriate sample size calculations for hypothesis testing), but does not directly evaluate health system barriers			Can assess health system barriers to attendance (e.g. vaccine stock-outs, health worker absence)	Yes, but only in the areas canvassed
